# Target metabolite and gene transcription profiling during the development of superficial scald in apple (*Malus x domestica* Borkh)

**DOI:** 10.1186/s12870-014-0193-7

**Published:** 2014-07-20

**Authors:** Nicola Busatto, Brian Farneti, Alice Tadiello, Urska Vrhovsek, Luca Cappellin, Franco Biasioli, Riccardo Velasco, Guglielmo Costa, Fabrizio Costa

**Affiliations:** 1 Department of Agricultural Sciences, Bologna University, Via Fanin 46, Bologna, 40127, Italy; 2 Research and Innovation Centre, Fondazione Edmund Mach, Via Mach 1, 38010 San Michele all’Adige, Trento, Italy

**Keywords:** Malus domestica, Cold storage, Postharvest, Superficial scald, 1-MCP, Polyphenol oxidase, Polyphenols, α-farnesene, Programmed death cell

## Abstract

**Background:**

Fruit quality features resulting from ripening processes need to be preserved throughout storage for economical reasons. However, during this period several physiological disorders can occur, of which superficial scald is one of the most important, due to the development of large brown areas on the fruit skin surface.

**Results:**

This study examined the variation in polyphenolic content with the progress of superficial scald in apple, also with respect to 1-MCP, an ethylene competitor interacting with the hormone receptors and known to interfere with this etiology. The change in the accumulation of these metabolites was further correlated with the gene set involved in this pathway, together with two specific VOCs (Volatile Organic Compounds), α-farnesene and its oxidative form, 6-methyl-5-hepten-2-one. Metabolite profiling and qRT-PCR assay showed these volatiles are more heavily involved in the signalling system, while the browning coloration would seem to be due more to a specific accumulation of chlorogenic acid (as a consequence of the activation of *MdPAL* and *MdC3H*), and its further oxidation carried out by a polyphenol oxidase gene (*MdPPO*). In this physiological scenario, new evidence regarding the involvement of an anti-apoptotic regulatory mechanism for the compartmentation of this phenomenon in the skin alone was also hypothesized, as suggested by the expression profile of the *MdDAD1*, *MdDND1* and *MdLSD1* genes.

**Conclusions:**

The results presented in this work represent a step forward in understanding the physiological mechanisms of superficial scald in apple, shedding light on the regulation of the specific physiological cascade.

## Background

Fruit quality is determined by a series of physiological modifications taking place throughout the maturation and ripening of fruit, starting from the initial phase of fruit development. These processes, which concern modification of the cell wall structure, accumulation in pigments, conversion of starch into sugar, the decrease in organic acid and flavour formation, are genetically coordinated in order to render the fruit more palatable to seed-dispersing organisms as well as more attractive for human consumption and diet [[1]-[3]]). Fruit ripening behaviour can also be divided into two classes (climacteric and non climacteric), according to the level of ethylene, a plant hormone fundamental for triggering and coordinating the processes leading to the final quality of fruit [[4],[5]]. To guarantee the maintenance of high quality standard, fruits are to date stored for a long time in an altered atmosphere, with the application of conditions such as low temperature or low oxygen concentration. These are non-physiological conditions interfering with physiological ethylene production and consequently with the natural progression of fruit ripening and senescence [[6]-[8]]. During postharvest storage, however, many disorders related to abiotic stress may arise due to chilling injuries or hypoxia [[9]-[14]].

Of the several postharvest disorders that can occur, superficial scald is one of the most important. In apple, the predominant scald symptom is represented by diffuse browning, generally limited to the skin and the underlying six cell layer [[15]]. This disorder normally occurs after the re-establishment of room temperature (20°C) following two or more months of cold storage (at -1 to 4°C; [[16]]). Superficial scald is a complex phenomenon influenced by environmental and genetic factors as well as the stage of fruit ripening. Specific apple cultivars, such as “Granny Smith”, “Fuji”, “Cripps Pink” and “Red Delicious” are, indeed, more susceptible than others [[17]]. Furthermore, ripe fruit is less susceptible to scald then immature fruit [[18]], and the green side of an apple is generally more prone to developing scald symptoms than the red one [[19]]. Despite the harmfulness and diffusion of this disorder, its etiology has still not been fully elucidated [[20]]. To date, the most investigated and accepted hypothesis about scald development is related to the accumulation of α-farnesene, an acyl sesquiterpene whose concentration increases during storage. This volatile is accumulated significantly in the skin, in particular in the external wax layer, due to its lipophilic characteristics [[19],[21],[22]]. Recent works have correlated the induction of superficial scald with the accumulation of α-farnesene autoxidation products, such as conjugated trienols (CTols), mainly 2,6,10-trimethyldodeca-2,7E,9,11-tetraen-6-ol [[23]]. The synthesis of α-farnesene is also supposed to be closely linked to the amount of ethylene [[24]], since this hormone modulates the expression of *MdAFS1* (α-farnesene synthase 1), the last gene in the α-farnesene biosynthetic pathway. The close connection between ethylene and α-farnesene was also further confirmed by the effect of the ethylene competitor 1-methylcyclopropene (1-MCP), which leads to a reduced accumulation of α-farnesene. 1-MCP is known to strongly influence the normal fruit ripening progression in climacteric fruit, due to its competing effect against ethylene at receptor level [[25]], as already documented in several genomic investigations carried out on apple, tomato and peach [[26]-[31]]. Beside this, 1-MCP has been recently also been widely used for the control of superficial scald in apple [[32]]. The complete mode of action of this compound in preventing scald is not yet fully clear, despite the fact that samples treated with 1-MCP showed a decreased expression of *MdAFS1* [[19],[33]-[35]]. Other recent references have instead indicated the initiation of free radical oxidation as the main factor of scald development in apple [[36]]. In this scenario, the autoxidation of α-farnesene could represent a side effect of a more complex and uncompleted process. In addition to the role of α-farnesene, there is another hypothesis about the oxidation of polyphenolic compounds, considered to be fundamental in generating of the browning of skin. These compounds, after the disruption of the cell inner membranes, interact with the polyphenol-oxidase enzyme (PPO) released from the chloroplast [[37]]. PPO thus turns polyphenols into oxidized forms, such as quinones [[38]-[40]]. Amines and thiol groups react with quinones, ultimately leading to the formation of brown pigments. This reduction has already been proposed by Boss *et al*. [[41]], who observed an upregulation of polyphenol oxidase transcripts in “Granny Smith” scalded tissues, while Piretti *et al*. [[42]] hypothesized that the brown pigmentation exhibited during scald was the final result of an oligomeric polyphenolic oxidation.

The effort of the scientific community to gain insight into superficial scald in apple, besides the understanding the basic physiological mechanisms governing this phenomenon, have mainly been focused on strategies designed to avoid this disorder [[43]-[45]]. Scalded fruit indeed has dramatic aesthetic deficiencies that can seriously compromise fruit marketability. To deal with this problem, several technological applications have been employed, such as forced ventilation, skin coating, heat shock and chemical treatment, in particular with DPA (diphenylamine [[46]]). However, while this amino antioxidant has been widely used untill now [[47]], its application is currently undergoing serious review, due to the possible risks associated with the compound. As an alternative molecule, postharvest management has taken advantage of 1-MCP, an ethylene inhibitor widely adopted to extend the storage capacity of climacteric fleshy fruits [[48],[49]]. However, most of the works presented so far in the scientific literature have been based mainly on enzymatic assay and the expression profile of a limited set of genes involved in this phenomenon, such as *AFS* and *PPO*.

In this work the change in the polyphenolic cascade was examined during the development of apple scald using a candidate gene qRT-PCR approach, together with targeted metabolic profiling. Finally, specific expression profiling based on different tissues suggested an intriguing evidence about the involvement of the programmed cell death (PCD) process, as a possible natural defence mechanism put into effect by the fruit to prevent the expansion of this postharvest disorder in apple. The results described and discussed in this work shed light on specific aspects of this disorder, helping to better clarify the physiological mechanisms taking place after harvest in apple fruit.

## Methods

### Plant materials and experimental design

Fruit were collected from “Granny Smith” apple trees planted in Faenza (Emilia-Romagna region, Italy), and maintained following standard agronomical practices in terms of mineral fertilisation, fruit thinning, canopy pruning and disease control. Apples were picked at the commercial harvest stage, instrumentally established at the I_AD_ value of 1.8-2. The I_AD_ is a non-destructive index of fruit ripening determined as the difference in absorbance between two wavelengths near the chlorophyll-a absorption peak (670 and 720 nm; [[50],[51]]). Homogeneous fruit (in terms of both ripening stage and shape) were sampled immediately after harvest (T0), and immediately divided into two batches. The first was treated with 1 ppm of 1-methyl-cyclopropene (1-MCP) for 24 hours, while the second was maintained as a control. The two sample batches were stored in cold room in normal atmospheric conditions at 0.5°C with 95% relative humidity. Samples from the two batches, were then removed from the cold room after one month (T1) and two months (T2) cold storage respectively. To further enhance scald development, additional apples were sampled three times during each period of cold storage, respectively after one (+1), four (+4) and eight (+8) days shelf life (room temperature of about 20°C; Additional file 1: Figure S1). To investigate symptom development throughout the fruit cortex, three types of tissues were assessed for each sample: skin (S, the peel and the underlying affected flesh), underskin (U, the few millimetres of flesh below the skin) and inner pulp (P).

### RNA isolation and qRT-PCR analysis

For each fruit, three tissues (skin, underskin flesh and pulp) were separately collected, cut in small pieces, immediately frozen in liquid nitrogen, ground into a fine powder and stored at -80°C until final processing. RNA extraction was performed using the Spectrum Plant total RNA kit (Sigma-Aldrich Co., St Luis, MO, USA). RNA was quantified using a NanoDrop ND-8000 spectrophotometer (Thermo Scientific, Waltham, MA, USA), while its purity and integrity was assessed with a 2100 Bioanalyzer (Agilent, Santa Clara, CA, USA). The RNA isolated was then converted into cDNA using the “SuperScript VILO cDNA Synthesis Kit” (Life Technologies, Carlsbad, CA, USA). Prior to this, 2 μg of total RNA from each sample was treated with 2 Units of Ambion rDNAse I (DNA free kit, Life Technologies, Carlsbad, CA, USA) and used as a starting template. Transcript quantification, carried out using the ViiA7™ instrument (Life Technologies, Carlsbad, CA, USA), was performed using the FAST SYBR GREEN MASTER MIX (Life Technologies, Carlsbad, CA, USA). PCR thermal conditions were: incubation at 95°C for 20 sec, 40 cycles of 95°C 1 sec and 60°C 20 sec. Finally, a cycle at 95°C for 15 sec, 60°C for 1 min and 95°C for 15 sec was applied to determine the melting curve. The Ct results were obtained by averaging two independent normalized expression values for each sample, carried out using the ViiA™ 7 Software (Life Technologies, Carlsbad, CA, USA) provided with the instrument. Relative gene expression was plotted as the mean of the normalized expression values using the Delta-Delta CT method [[52]] and Md8283 was employed as housekeeping gene [[53],[54]].

### Gene identification and primer design

The gene set investigated in this survey was selected on the basis of the polypohenolic pathway as described by Kirk et al. [[55]]. Since a strong correlation has already been observed between the metabolism of polyphenolic compounds and a similar phenotype in apple (flesh browning [[40]]), we focused our efforts on comprehension of polyphenolic pathway regulation. The gene ID was retrieved from studies already published [[56]-[62]] for each member responsible for this physiological biosynthetic pathway, while cDNA sequences were in silico retrieved from the NCBI database (http://www.ncbi.nlm.nih.gov/). The ORF portion was defined from each sequence, in order to characterize the CDS from the UTR portions, when possible. To target the corresponding specific apple element, each single CDS sequence was blasted on the apple geneset available in the Rosaceae database (www.rosaceae.org). Gene IDs with the highest E-value and score were selected as candidates. For each gene, specific primer pairs were designed on the flanking regions of each MDP, isolated by aligning the UTR on the cDNA sequence, in order to define unique target elements for each polyphenolic biosynthetic gene. In addition to these, the expression profile of other five genes was assessed. The polyphenol oxidase gene *MdPPO* (MDP0000699845) was retrieved by Di Guardo et al. [[40]], while *MdAFS1*, involved in the biosynthetic pathway of α-farnesene, was selected by blasting the cDNA sequence designed in Lurie et al. [[33]] on the apple genome following the procedure previously described. Finally, three genes (*MdDAD1*, *MdDND1* and *MdLSD1*) involved in anti-apoptosis mechanism were also considered. The cDNA sequence of *MdDAD1* was obtained from Dong et al. [[63]] and used a BLAST query in order to identify the corresponding MDP in the geneset mentioned above. *DND1* [[64]] and *LSD1* [[65]] were selected as candidate anti-apoptotic genes due to the function previously characterized in *Arabidopsis thaliana*. The cDNA sequences of these elements were initially retrieved from the Arabidopsis database (www.arabidopsis.org), and further blasted on the apple geneset (Additional file 2: Figure S2). Gene predictions with the highest E-value and score were chosen as candidate homologues in apple.

### Polyphenolic compound extraction and separation

Phenols were extracted and analysed from the ground tissues of “Granny Smith” apples following the procedure reported in Theodoridis et al. [[66]] and Di Guardo et al. [[40]]. Each sample was represented by three biological replicates. 2 g of powdered tissues were extracted in sealed glass vials using 4 mL of water/methanol/chloroform solution (20:40:40). After vortexing for 1 min, the samples were mixed using an orbital shaker for 15 min at room temperature, and further centrifuged at 1000 g (4°C) for 10 min, after which the upper phases, made up of aqueous methanol extract, were collected. Extraction was repeated by adding another 2.4 mL of water/methanol (1:2) to the pellet and chloroform fractions. After the final centrifugation, the upper phases from the two extractions were combined and brought to the volume of 10 mL and filtered with a 0.2 μm PTFE filter prior to liquid chromatography-mass spectrometry analysis. Ultraperformance liquid chromatography was performed employing a Waters Acquity UPLC system (Milford, MA, USA) coupled to a Waters Xevo TQMS (Milford, MA, USA) working in ESI ionisation mode [[67]]. Separation of the phenolic compounds was achieved on a Waters Acquity HSS T3 column 1.8 μm, 100 mm × 2.1 mm (Milford, MA, USA), kept at 40°C, with two solvents: A (water containing 0.1% formic acid) and B (acetonitrile containing 0.1% formic acid). The flow was 0.4 mL/min, and the gradient profile was 0 min, 5% B; from 0 to 3 min, linear gradient to 20% B; from 3 to 4.3 min, isocratic 20% B; from 4.3 to 9 min, linear gradient to 45% B; from 9 to 11 min, linear gradient to 100% B; from 11 to 13 min, wash at 100% B; from 13.01 to 15 min, back to the initial conditions of 5% B. A volume of 2 μL from both standard solutions and samples was injected, after which the needle was rinsed with 600 μL of weak wash solution (water/methanol, 90:10) and 200 μL of strong wash solution (methanol/water, 90:10). Samples were kept at 6°C during mass spectrometry detection, performed with a Waters Xevo TQMS (Milford, MA, USA) instrument equipped with an electrospray (ESI) source. Capillary voltage was 3.5 kV in positive mode and −2.5 kV in negative mode; the source was kept at 150°C; desolvation temperature was 500°C; cone gas flow, 50 L/h; and desolvation gas flow, 800 L/h. Unit resolution was applied to each quadrupole. Data were processed using Waters MassLynx 4.1 and TargetLynx software.

135 phenolic compounds were initially selected for the quantitative measurement assay [[67]]. The choice of the metabolites was mainly based on their importance and/or relevance for food quality, covering the major classes. In particular, benzoates, phenylpropanoids, coumarins, stilbenes, dihydrochalcones, and flavonoids commonly occurring in plants were included, together with metabolites specific to a single species or family. Stock solutions of each individual standard solution were prepared in pure methanol. These starting solutions were used to prepare 16 standard mixtures including 6 − 10 compounds each. Serial dilutions were prepared to obtain 24 lower concentrations (dilution factors of 1 − 60000) for linear dynamic range assessment.

### VOC characterization using PTR-ToF-MS

Measurements of VOCs in apple tissues were performed as follows. 2.5 g of powdered frozen tissue were immediately inserted into a 20 mL glass vial equipped with PTFE/silicone septa (Agilent, Santa Clara, CA, USA) and mixed with 2.5 mL of deionized water, 1 g of sodium chloride, 12.5 mg of ascorbic acid, and 12.5 mg of citric acid, and then preserved at 4°C untill the assessment. Analysis was performed on three replicates using commercial PTR-ToF-MS 8000 apparatus (Ionicon Analytik GmbH, Innsbruck, Austria). The conditions in the drift tube were: 110°C drift tube temperature, 2.25 mbar drift pressure, 550 V drift voltage. This leads to an E/N ratio of about 140 Townsend (Td), with E corresponding to the electric field strength and N to the gas number density (1 Td = 10 − 17 Vcm2). The sampling time per channel of ToF acquisition was 0.1 ns, amounting to 350,000 channels for a mass spectrum ranging up to m/z = 400. Each measurement was conducted automatically after 20 minutes of sample incubation at 40°C by using an adapted GC autosampler (MPS Multipurpose Sampler, GERSTEL) and it lasted for about 2 minutes. During measurements 100 sccm of zero air was continuously injected into the vial, through a needle heated to 40°C; the outflow was instead delivered via Teflon fittings to the PTR-Tof-MS through a second heated needle (40°C). The analysis of PTR-ToF-MS spectral data proceeded as follows. Count losses due to the ion detector dead time were corrected off-line through a Poisson statistics based method [[68]], while internal calibration was performed according to the procedure described in Cappellin et al. [[69]]. This approach makes it possible to reach a mass accuracy higher than 0.001 Th, which is sufficient for sum formula determination in our case. Compound annotation was carried out following comparison of spectra with the fragmentation data of compound reference standards. Noise reduction, baseline removal and peak intensity extraction were performed by using modified Gaussians to fit the peaks. Absolute headspace VOC concentrations expressed in ppbv (parts per billion by volume) were calculated from peak intensities according to the formula described by Lindinger et al. [[70]]. A constant reaction rate coefficient of 2∙10-9 cm3/s was used in the calculations, introducing a systematic error of up to 30% that can account for the actual rate if the coefficient is known [[71]].

## Results and discussion

### Superficial scald development in “Granny Smith” and the polyphenolic pathway

**Figure 1:**
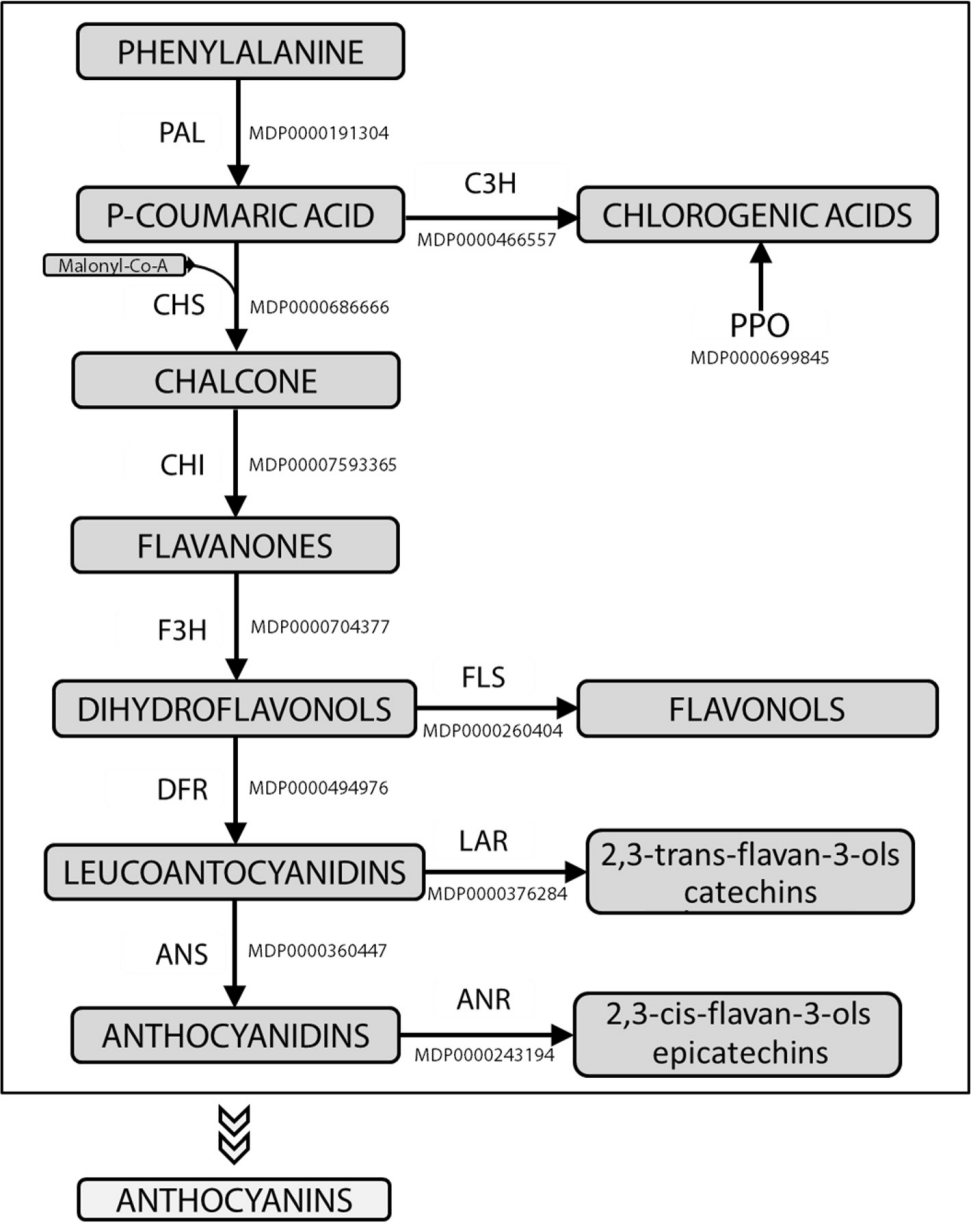
**Visual representation of the polyphenolic pathway in apple.** The main polyphenolic classes (adjusted form Henry-Kirk *et al.* [[55]]) are highlighted in grey boxes. For each step the committed enzyme is indicated with the corresponding MDP gene ID, whose expression was experimentally assessed. Outside the main frame anthocyanins are also indicated, although they were not investigated, since this class (leading to the red coloration) is negligible in the “Granny Smith” apple cultivar.

To study the accumulation and progression of superficial scald development, fruit from the “Granny Smith” apple cultivar were divided in two groups immediately after harvest, one group being kept as a control while the second was treated with 1-MCP, an ethylene competitor [[72]] known to interact with scald development in apple [[73]]. The physiological progression of the disorder was monitored by sampling apples at two storage periods (one and two months respectively), as well as three times during eight days shelf life, as shown in Additional file 1: Figure S1. Following visual inspection, browning in scalded apples occurred only in the skin of T2 stage control samples, with an increasing magnitude over the week of shelf life (Additional file 3: Figure S3). For each sample included in this experimental design, three tissues were specifically isolated, skin, underskin, and inner flesh (pulp), in order to verify the spatial response of the fruit to this phenomenon, in terms of gene expression and secondary metabolite variation. The pathway investigated here considered the biochemical cascade of phenylamine, which leads to the synthesis of polyphenols, such as chlorogenic acid, flavonols, flavan-3-ols (catechin and epicatechin), as illustrated in Figure 1. Expression of 11 structural genes (Additional file 4: Table S1) was located and analyzed along this pathway. Of these six (*MdPAL*, *MdCHS*, *MdCHI*, *MdF3H*, *MdDFR* and *MdANS*) were designated to the main central cascade. Furthermore, *MdC3H*, *MdFLS*, *MdLAR* and *MdANR* were selected because of their essential role in the formation of four distinct major classes of polyphenolic compounds: chlorogenic acids, flavonols, epicatechin and catechin, respectively. Moreover, one member of the *PPO* family was also investigated (MDP0000699845) as responsible for the transformation of polyphenolic compounds into oxidized brownish forms.

### The candidate gene expression profile and polyphenol quantification showed chlorogenic acid is the major determinant in superficial scald development

The polyphenol content was quantified for all the samples, characterizing five major compounds namely chlorogenic acid, phlorizin, flavonols, catechin and epicatechin. In a general overview, the total polyphenolic accumulation (Additional file 5: Figure S4 and Additional file 6: Table S2) was clearly higher in the skin as compared to the other two tissues (underskin and pulp) with about a 5 fold of change. This trend is also confirmed by the different accumulation of phlorizin, flavonols and epicatechin, but not catechin. According to previous studies [[74]-[76]] “Granny Smith” fruit often showed a lower concentration of catechin than epicathechin, especially in the skin, where the amount of epicatechin is approximately double.

**Figure 2:**
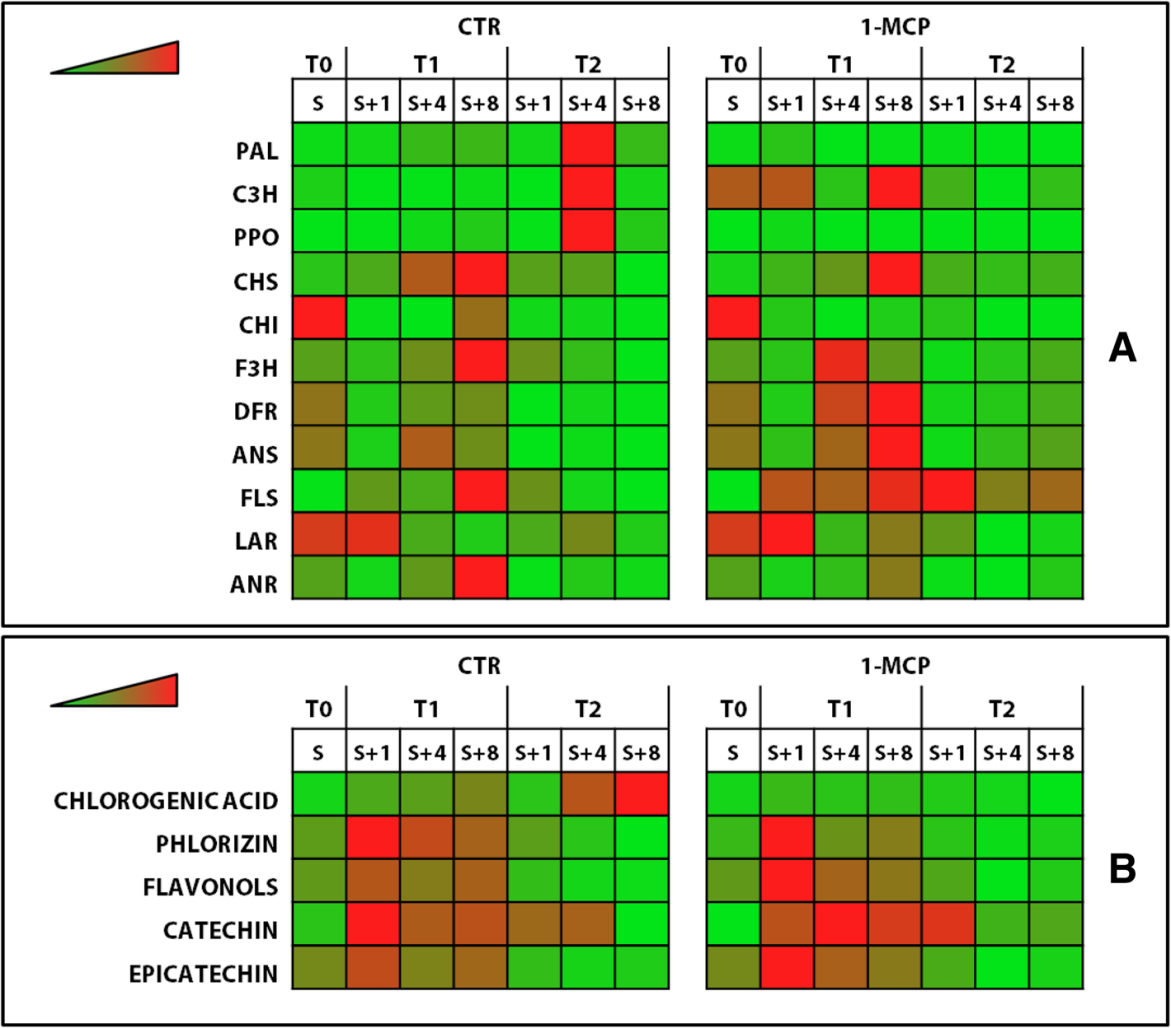
**Gene expression and secondary metabolite heat-map profile in skin tissue.** Gene transcript dynamics are shown in **panel A**, while **panel B** shown the accumulation of secondary metabolites in the control (CTR, showing scald) and 1-MCP treated sample (in which this disorder was prevented). For both panels, data spanned from light green (low intensity) to light red (high intensity), as illustrated by the color scale. The data plotted on panel “A” are expressed as normalized expressions, whereas panel “B” shows μg/g of fresh weight (FW). The full gene expression and metabolite profile for the entire set of tissues are shown in Figures 3 and 5 as well as in Additional file 5: Figure S4 and Additional file 6: Table S2.

Examining each category, a specific regulation over the period of storage was also observed. In phlorizin, for instance, the accumulation in the samples collected after two months of storage (T2) was lower than after one month (T1), while for flavonols an opposite trend was detected (Additional file 5: Figure S4). In addition to this, for some compounds (such as phlorizin, assessed in underskin and pulp collected at T1, and flavonol at T1 and T2_S+1_ in skin and underskin) a reduced accumulation was also detected after treatment with 1-MCP. This general polyphenolic accumulation (Figure 2B) was compared with the transcript profile of the eleven genes selected to represent the biosynthetic pathway of these metabolites (Figure 2A). From the general heatmap, illustrating both the expression profile and the polyphenolic accumulation in the skin alone (tissue concerned by superficial scald), it is worth noting the different transcriptomic pictures in the control and treated samples. This particular response after treatment highlighted how 1-MCP is used to turn off fundamental genes involved in the ripening pathway, modifying gene expression and detecting three main transcriptome dynamics. In the first group three genes, *MdPAL*, *MdPPO* and *MdC3H*, specifically expressed at the T2_S+4_ stage (coinciding with scald development), were completely down-regulated by 1-MCP, suggesting possible positive regulation by ethylene. In the specific case of the last gene, *MdC3H*, designated to controlling the final synthesis of the chlorogenic acid, the application of 1-MCP slightly anticipated its mRNA accumulation from the T2 to the T1 stages. The second group, represented by the three genes (*MdCHS*, *MdCHI* and *MdF3H*) involved in the cascade from *p*-coumaric acid to dihydroflavonols (step before the branching to flavonols), together with *MdANR* (involved in the final conversion of flavan-3-ols from anthocyanidins), showed a reduction in transcript accumulation after the application of the ethylene competitor 1-MCP. Finally the expression level of the last set of genes (*MdDFR*, *MdANS*, *MdFLS* and *MdLAR*), located downstream of the polyphenolic pathway, was instead slightly enhanced by the application of 1-MCP (Figure 2 and Additional file 7: Figure S5).

**Figure 3:**
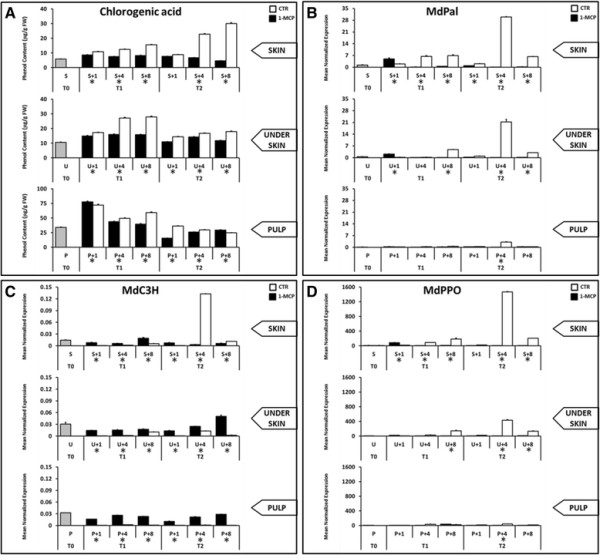
**Physiological regulation of the chlorogenic acid over time and in tissues and treatments. Panel “A”** shows the accumulation of this secondary metabolite in μg/g of FW. The other three panels **(B, C and D)** instead illustrate the expression profile of the three genes involved in the chlorogenic acid pathway, namely *MdPAL*, *MdC3H* and *MdPPO,* and its oxidation process. Gene expression is visualized on the basis of Mean Normalized Expression. The bar on the histogram represents the standard error for each panel. Asterisk indicates a difference statistically significant based on a LSD-ANOVA (*P-*value ≤ 0.05).

Although superficial scald is a phenomenon which mainly concerns fruit skin, further investigation of the possible progression in inner tissue was assessed. In particular, we aimed to unravel the response mechanism limiting browning progression. The different categories of polyphenolic compounds were differently accumulated in the samples defined in the experimental design planned here. In particular, phlorizin, catechin and epicatechin decreased constantly throughout storage and shelf life, while flavonols showed contrasting dynamics, as they were instead accumulated (Additional file 5: Figure S4). The most interesting profile was observed for the chlorogenic acid, which was accumulated in the skin tissue from T1 to T2 (as well as over the shelf life progression), while in underskin and pulp tissues it showed a decreasing trend during the different stages. The higher accumulation of chlorogenic acid (Figure 3A) coincided with the scald burst occurring in the T2_S+4_ stage, which was also characterized by the highest expression level of the genes involved in the chlorogenic acid pathway, namely *MdPAL*, *MdC3H* and *MdPPO* (Figure 3B, C and D). The increased production of chlorogenic acid during scald, accompanied by the activation of *PAL* and *C3H*, can be though to be an antioxidant protection system activated by the fruit during storage. The appearance of superficial scald in “Granny Smith” apples is indeed often positively correlated with the accumulation of reactive oxygen species in the damaged tissues [[36],[77]], probably caused by the negative effect of cold storage on the fluidity and integrity of cell membranes [[78]], leading to ion leakage and to general decompartmentation. This phenomenon related to chilling injuries, has been observed for apple, as well as for other fruit species [[10],[79]].

In this scenario, browning coloration may occur as a response to the activation of *MdPPO* (which uses chlorogenic acid as the main substrate), to maintain the system in a state of physiological equilibrium, as also observed in the case of internal browning [[40]], a similar process resulting in the induction of *MdPAL* and *MdPPO*. In contrast to internal browning, superficial scald was more localized in the cell of the skin layer. This tissue specificity, already highlighted by the polyphenolic analysis, is also supported by specific gene expression. *MdPAL*, MdC3H and *MdPPO* are indeed mainly expressed in the skin rather than in the underskin and pulp, as compared to the other genes involved in the polyphenolic pathway (Figure 3 and Additional file 7: Figure S5). The specific accumulation of both chlorogenic acid and relative gene transcripts suggest that this compound and subsequent activation of *MdPPO* gene are the main events in the development of the disorder. This hypothesis is also experimentally supported by comparison with the samples treated with 1-MCP. This compound, known to interfere with scald development during postharvest storage [[80]], effectively blocked the synthesis of chlorogenic acid in the skin (Figure 3A). In parallel, 1-MCP strongly and specifically downregulated the expression of *MdPAL*, *MdC3H* and *MdPPO* (Figure 3B, C and D). The fact that the application of the ethylene competitor was not equally effective in the other genes located in the polyphenolic pathway (Additional file 7: Figure S5) supported the role of chlorogenic acid as the fundamental physiological pathway concerned during browning. It is also interesting to note that the effect of 1-MCP, particularly in the T2 stages (affected by the incidence of skin browning), was less evident in the inner tissues (underskin and pulp). The expression level of *MdC3H* was basically unchanged in the underskin and pulp tissues (Figure 3C), suggesting that scald in apple focuses on the skin because of specific metabolic regulation of these classes of secondary metabolites in different fruit tissues, in response to localized chilling injury. It is also worth noting two inconsistencies observed between gene expression level and metabolite accumulation. The first was represented by the peak in expression of *MdPAL* at T2_U+4_, which does not correlate with the unchanged accumulation of chlorogenic acid. This trend can however be explained by the low expression of *MdC3H*, suggesting the incomplete biosynthesis of chlorogenic acid, this gene representing this gene the final step in this cascade. Another incongruence regarded the high accumulation of this compound in the pulp, despite a low gene activity in the T1 stages. This situation can be explained by an accumulation of chlorogenic acid during the first month of cold storage, after which it showed continuous and linear degradation, thus not requiring the activation of this biosynthetic pathway for the synthesis of new chlorogenic acid.

### The role of α-farnesene and CTols during scald development in apple

In addition to the polyphenolic characterization, further investigation of the role of α-farnesene and its oxidative product 6M5H2one (6-methyl-5-hepten-2-one) was also carried out. α-farnesene has been indicated to date as the main compound involved in scald development [[81]]. In particular, it is the oxidative products of α-farnesene that have been suggested to be the causal agent of this disorder, since external application of CTols on the skin of “Granny Smith” apples induced the development of scald-like symptoms [[23]]. To verify the role of α-farnesene and 6M5H2one in the process, these two volatile organic compounds (VOCs) were measured using a PTR-ToF-MS, the new version of a mass spectrometer based on a proton transfer reaction [[82]].

**Figure 4:**
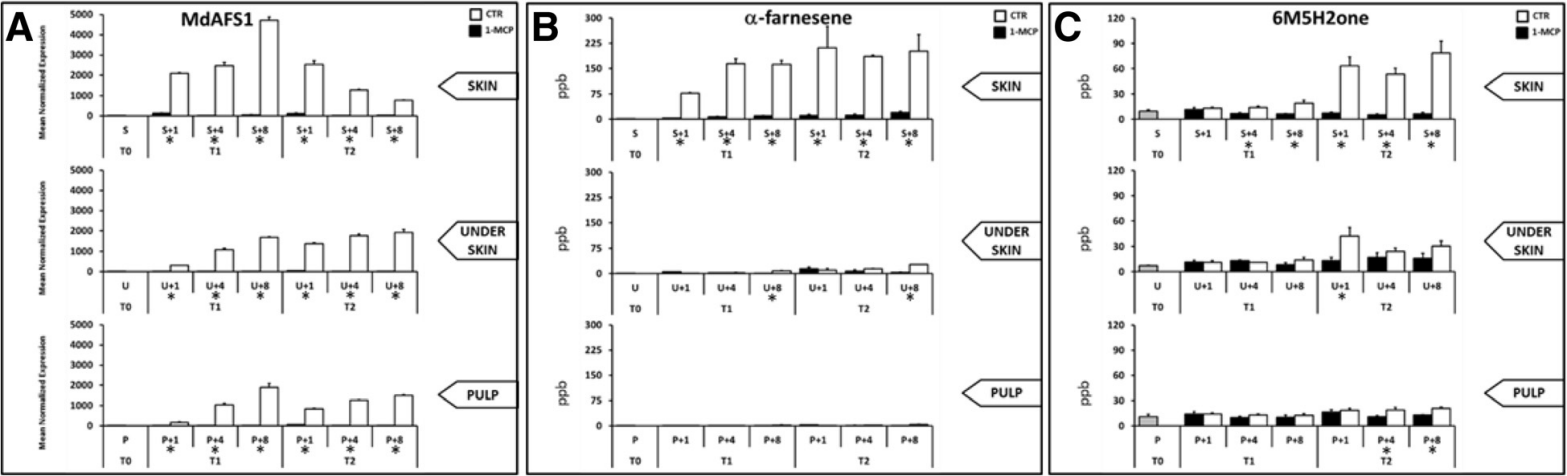
**MdAFS1 expression analysis together with α-farnesene and 6M5H2one profiling using PTR-ToF-MS.** The expression profile of *MdAFS1*, the gene responsible for α-farnesene biosynthesis, is shown in **panel A**, while assessment of the metabolites concentration (in ppb) of α-farnesene and 6M5H2one is shown in **panel B and C**, respectively. Asterisk indicates a difference statistically significant based on a LSD-ANOVA (*P-*value ≤ 0.05).

According to other references [[16],[19],[20],[33]] α-farnesene was linearly accumulated after the first month of cold storage and in particular during the first period of room temperature shelf life, with a clear increase from the T1_+1_ to T1_+8_ stages, after which a more constant profile was observed during T2 shelf life (Figure 4B). The burst in this volatile was positively correlated with the activation and transcription dynamics of *MdAFS1*, the gene designated to encoding the last step of the α-farnesene synthesis (Figure 4A). *MdAFS1* increased its mRNA level from T1_S+1_ to T1_S+8_, to then decrease in the T2 stages. Volatile concentration and *MdAFS1* expression were significantly affected by 1-MCP, since its application reduced both VOCs and *MdAFS1* accumulation almost completely (Figure 4A, C and B). Beside α-farnesene, PTR-ToF-MS also efficiently detected 6M5H2one, a volatile ketone produced through oxidation of α-farnesene. Rowan et al. [[23]] suggested that the concentration of 6M5H2one resulting from autoxidation of α-farnesene, could be considered to be a reliable indicator of oxidation in apple peel, since the autoxidation of α-farnesene indicates a free-radical-mediated reaction, involved in the production of CTols [[20],[83]].

Interestingly, the accumulation of 6M5H2one occurred after its precursor. 6M5H2one, indeed remained at basal concentration as compared to harvest, throughout the T1 stages, after which its accumulation increased considerably during the T2 stages (Figure 4C), coinciding with the development of scald symptoms. The time-wise regulation of these two VOCs suggests that rather than being the causal event of scald, α-farnesene and 6M5H2one play a more regulatory role. In this scenario, we can surmise that α-farnesene is accumulated in apple skin during postharvest storage. Then, in response to oxidative stress (one of the possible cause stimulating the development of scald) the α-farnesene may be auto-oxidized in CTols, which then act as a signaling molecule, triggering a defence mechanism based on antioxidant protection, carried out by an increased accumulation of polyphenolic compounds, in particular chlorogenic acid. To maintain the system with feedback regulation, the excess of chlorogenic acid is then oxidized by the polyphenolic oxidase enzyme, generating the consequent brown coloration.

### An anti-apoptosis system can activate a defence mechanism against scald progression in apple

In addition to the genes involved in the polyphenolic cascade, three elements involved in the apoptosis mechanism were also investigated, namely *MdDAD1*, *MdDND1* and *MdLSD1*.

The involvement of programmed cell death (PCD) activity in the progress of post-harvest disorders in apple has recently been suggested [[84]], hypothesizing a possible role for PCD, in synergy with oxidative stress and ethylene, as part of a series of processes leading to superficial scald, internal browning and bitter pit symptoms. To the best of our knowledge, very little data about PCD in apple have been presented till now, and in most cases they were related to stress responses in the suspension of cell cultures [[85],[86]]. To date, the only clear evidence about the presence of the PCD mechanism during apple storage is represented by the activity of *DAD1*. As reported by Dong et al. [[63]], *MdDAD1* is an apple element encoding for a subunit of the mammalian oligosaccharyltransferase homolog (defender against cell death 1 - *DAD1*), whose expression gradually rises during ripening at room temperature. *DND1* in Arabidopsis (AT5G15410) instead encodes a functional cyclic nucleotide-gated cation channel directly involved in the pathogen induced Ca^2+^ influx (*CNGC2*) activated during hypersensitive responses [[64]]. Interestingly, a high Ca^2+^ concentration in the fruit is an important pre-harvest parameter for the reduction of superficial scald incidence [[87],[88]], as well as an ubiquitous signal in abiotic stress resistance, such as cold tolerance [[89]], and PCD [[84],[90]]. Finally, *AtLSD1* (AT4G20380) encodes a C_2_H_2_ zinc finger transcription factor that monitors a superoxide-dependent signal, which negatively regulates PCD in plants [[65]]. This gene indeed represents an intriguing connection between ROS-associated signalling, low temperature-dependent PCD and cold stress tolerance [[91]], and it is thought to limit cell death via up-regulation of Cu-Zn-superoxide dismutase acting as protection against uncontrolled oxidative processes during chilling injuries [[92]].

**Figure 5:**
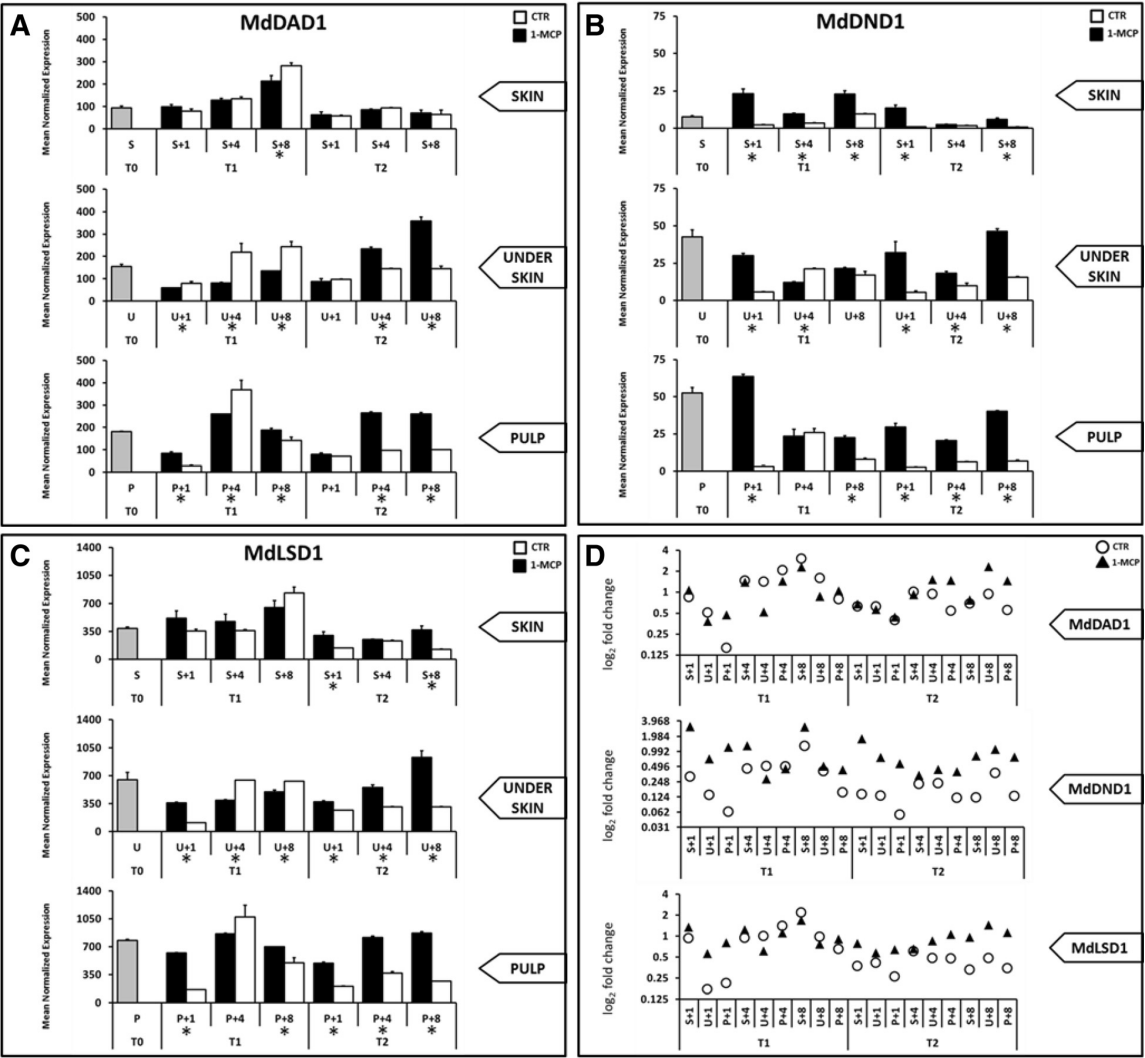
**Expression profile of the three genes involved in the anti-apoptotic mechanism.** Mean normalized expression of *MdDAD1***(panel A)**, *MdDND1***(panel B)** and *MdLSD1***(panel C)** in the three apple tissues over time and comparison between the control (CTR) and treated (1-MCP) samples. The standard error is also reported for each bar. **Panel “D”** is instead shows the log_2_ fold change of the expression profile of these three genes anchored to the respective T0 stage. Asterisk indicates a difference statistically significant based on a LSD-ANOVA (*P-*value ≤ 0.05).

From the transcriptomic profiles of the three apple orthologs (Figure 5A, B and C), it is evident that starting from the re-establishment of room temperature, and only after two months of cold storage, the mRNA accumulation in the skin was lower as compared to the underskin and fruit pulp. This regulation was not observed in T1 stages, suggesting this difference was properly regulated by the occurrence of superficial scald disorder. The high gene expression in the two tissues not affected by the brown coloration, as compared to the skin, was also magnified by treatment with 1-MCP (Figure 5D), suggesting the involvement of an anti-apoptotic mechanism as a protective system against scald. The anti-apoptotic system may thus act in connection with the oxidation of the chlorogenic acid as a self-defence mechanism. Following the signal released by the oxidation of α-farnesene and the high accumulation of polyphenolic compounds, further protection of the underlying tissue may be initiated by the activation of these genes, which only contribute towards isolating scald progression in the skin of apple. It is interesting to note that a similar mechanism was observed in walnut transgenic line [[93]]. The silencing of *PPO* leads to an over-accumulation of tyramine and polyphenolic substrate, with a corresponding decrease in their reaction products, underlying the possible connection between *PPO* activity, polyphenols metabolism and PCD events.

## Conclusion

The results described in this work shed light on the physiological process leading to scald development in apple, a harmful postharvest disorder affecting the marketability of specific apple cultivars. The analysis performed on the main polyphenolic cascade highlighted the pathway of chlorogenic acid, and its subsequent oxidation by polyphenoloxidase, as the major factor responsible for scald symptoms. In addition to this, we propose a new role for α-farnesene and its oxidative product (CTols), as a triggering signal for browning. Finally, we also surmise the involvement of an anti-apoptotic regulation in order to prevent the diffusion of this disorder in the inner tissues of apple.

## Abbreviations

*PAL*: Phenylalanine ammonia lyase

*CHS*: Chalcone synthase

*CHI*: Chalcone isomerase

*MdF3H*: Flavone 3-hydroxylase

*DFR*: Dihydroflavonol 4-reductase

*ANS*: Anthocyanidin synthase

*C3H*: p-coumarate 3-hydroxylase

*FLS*: Flavonol synthase

*LAR*: Leucoanthocyandin reductase

*ANR*: Anthocyanidin reductase

*PPO*: Polyphenol oxidase

*AFS1*: α-farnesene synthase

*DAD1*: Defender against cell death 1

*DND1*: Defense no death 1

*LSD1*: Lesion simulating disease resistance 1

1-MCP: 1-methyl-cyclopropene

CTols: Conjugated trienols

6M5H2one: 6-methyl-5-hepten-2-one

PCD: Programmed cell death, Md, *Malus domestica*

At: *Arabidopsis thaliana*

## Competing interest

The authors declare that they have no competing interest.

## Authors’ contributions

NB performed gene expression analysis and drafted the manuscript, AT supported the molecular work, BF performed metabolite and VOC analysis and contributed towards drafting the manuscript, UV interpreted the metabolite data, LC and FB interpreted the PTR-ToF-MS data, RV and GC supported the project, FC designed the experiment, coordinated the work and finished the manuscript. All authors read and approved the final manuscript.

## Additional files

## Supplementary Material

Additional file 1: Figure S1. Experimental design. After harvest (T0), the apples were divided into two batches. The first was considered as a control (CTR), while the second was treated with 1-methyl-cyclo-propene (1-MCP). Both subsets were placed in a cold storage room (+0.5°C) for one and two months respectively. After cold storage, the treated and untreated fruit were placed at room temperature and sampled after one (+1), four (+4) and eight (+8) days. 7 stages were thus defined (T0: harvest, T1_+1_: 1 month of cold storage + 1 day of shelf life, T1_+4_: 1 month of cold storage + 4 days of shelf life, T1_+8_: 1 month of cold storage + 8 days of shelf life, T2_+1_: 2 months of cold storage + 1 day of shelf life, T2_+4_: 2 months of cold storage + 4 days of shelf life, T2_+8_: 2 months of cold storage + 8 days of shelf life). Moreover, each stage was repeated in three different tissues: S: skin, U: underskin and P: pulp. Click here for file

Additional file 2: Figure S2. Sequence alignment of *DND1*, *DAD1* and *LSD1*. Sequence alignment between the Arabidopsis protein sequences of *DND1*, *DAD1* and *LSD1* with their putative apple orthologs. The conserved residues are shown with black frames. Click here for file

Additional file 3: Figure S3. Superficial scald development in “Granny Smith” apples. Representative pictures of superficial scald evolution in the control (CTR) and treated samples (1-MCP), after one (panel from A to F) and two months (panel from G to N) cold storage. Panel O shows a portion of scalded peeled apple tissue, at the T2 + 8 stage, highlighting isolation of the brown coloration in the skin alone. Click here for file

Additional file 4: Table S1. List of primers. List of all the primer pairs used in this work. The gene name, the annotation, the gene ID according to the code used in the apple genome database (www.rosaceae.org), the chromosome on which the gene is located, primer sequences designed for qRT-PCR analysis and the relative references are indicated for each pair. Click here for file

Additional file 5: Figure S4. Polyphenolic compounds characterization. Characterization of four major classes of polyphenolic compounds, namely phlorizin, flavonols, catechin and epicatechin. The last panel instead shows the general phenolic accumulation profile, including all the categories investigated in this study. The amount of each compound is expressed as μg/g of fresh weight (FW). The standard error is also reported for each bar. Asterisk indicates a difference statistically significant based on a LSD-ANOVA (*P-*value ≤ 0.05). Click here for file

Additional file 6: Table S2. Table of polyphenol compounds quantified in this work. The amount of each compound is expressed as μg/g of fresh weight (FW). Each sample was represented by three separated biological replicates. Click here for file

Additional file 7: Figure S5. Expression profile of genes involved in polyphenolic biosynthesis. Expression profile of all other genes participating in polyphenolic biosynthesis and not presented in the main text, namely *MdCHS*, *MdCHI*, *MdF3H*, *MdDFR*, *MdANS*, *MdFLS*, *MdLAR* and *MdANR*. The standard error is also reported for each bar. Asterisk indicates a difference statistically significant based on a LSD-ANOVA (*P-*value ≤ 0.05). Click here for file
