# Supplementary figures and images for: Target metabolite and gene transcription profiling during the development of superficial scald in apple (Malus x domestica Borkh)
Source: BMC Plant Biol. 2014 Jul 20;14:193. doi: 10.1186/s12870-014-0193-7 (PMC4115171; doi:10.1186/s12870-014-0193-7)

## Slide 1
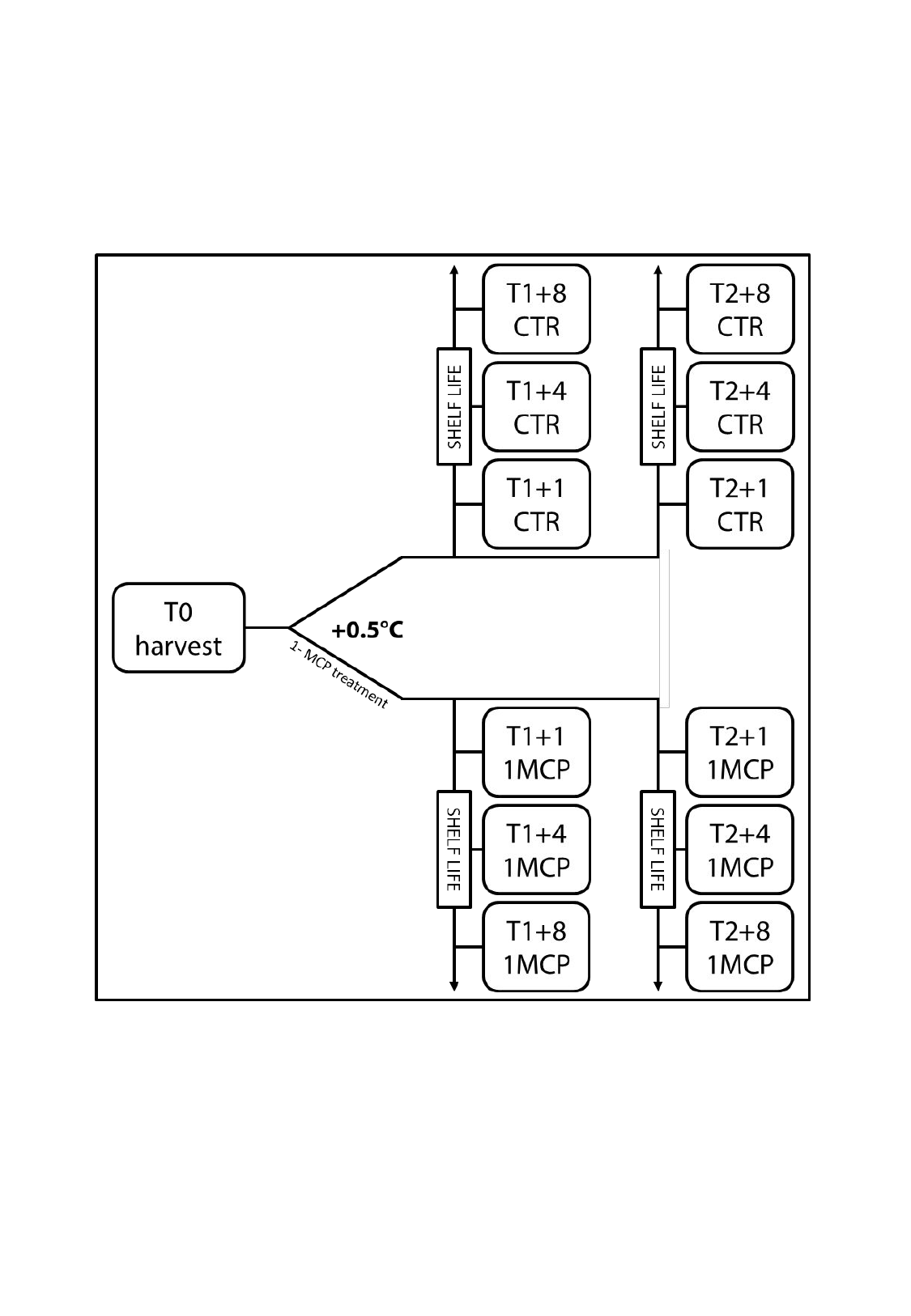

Supplement: Additional file 1: Figure S1. — Experimental design. After harvest (T0), the apples were divided into two batches. The first was considered as a control (CTR), while the second was treated with 1-methyl-cyclo-propene (1-MCP). Both subsets were placed in a cold storage room (+0.5°C) for one and two months respectively. After cold storage, the treated and untreated fruit were placed at room temperature and sampled after one (+1), four (+4) and eight (+8) days. 7 stages were thus defined (T0: harvest, T1+1: 1 month of cold storage + 1 day of shelf life, T1+4: 1 month of cold storage + 4 days of shelf life, T1+8: 1 month of cold storage + 8 days of shelf life, T2+1: 2 months of cold storage + 1 day of shelf life, T2+4: 2 months of cold storage + 4 days of shelf life, T2+8: 2 months of cold storage + 8 days of shelf life). Moreover, each stage was repeated in three different tissues: S: skin, U: underskin and P: pulp. [file s12870-014-0193-7-S1.pptx]

## Slide 1
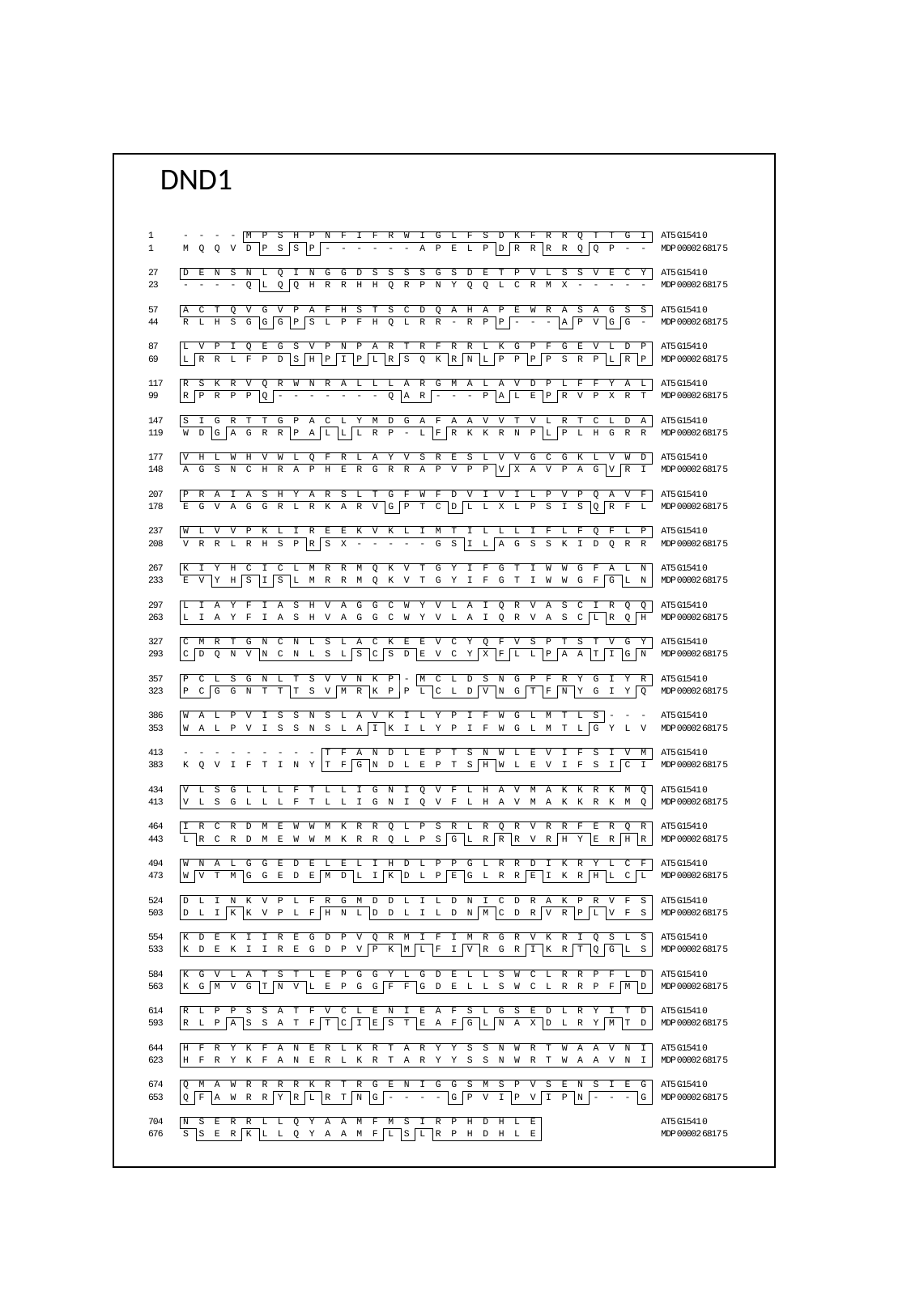

DND1

## Slide 2
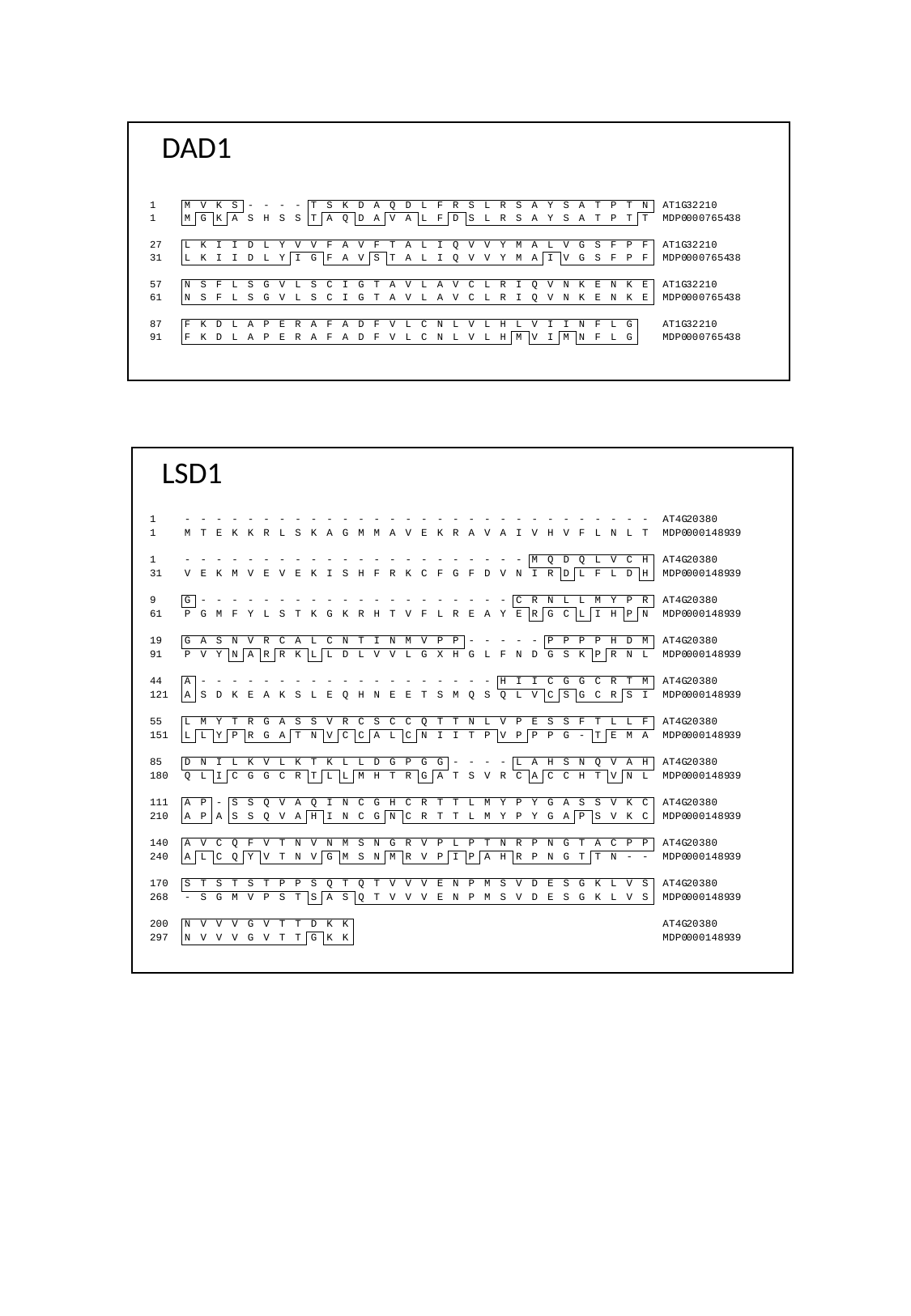

DAD1
LSD1

Supplement: Additional file 2: Figure S2. — Sequence alignment of DND1, DAD1 and LSD1. Sequence alignment between the Arabidopsis protein sequences of DND1, DAD1 and LSD1 with their putative apple orthologs. The conserved residues are shown with black frames. [file s12870-014-0193-7-S2.pptx]

## Slide 1
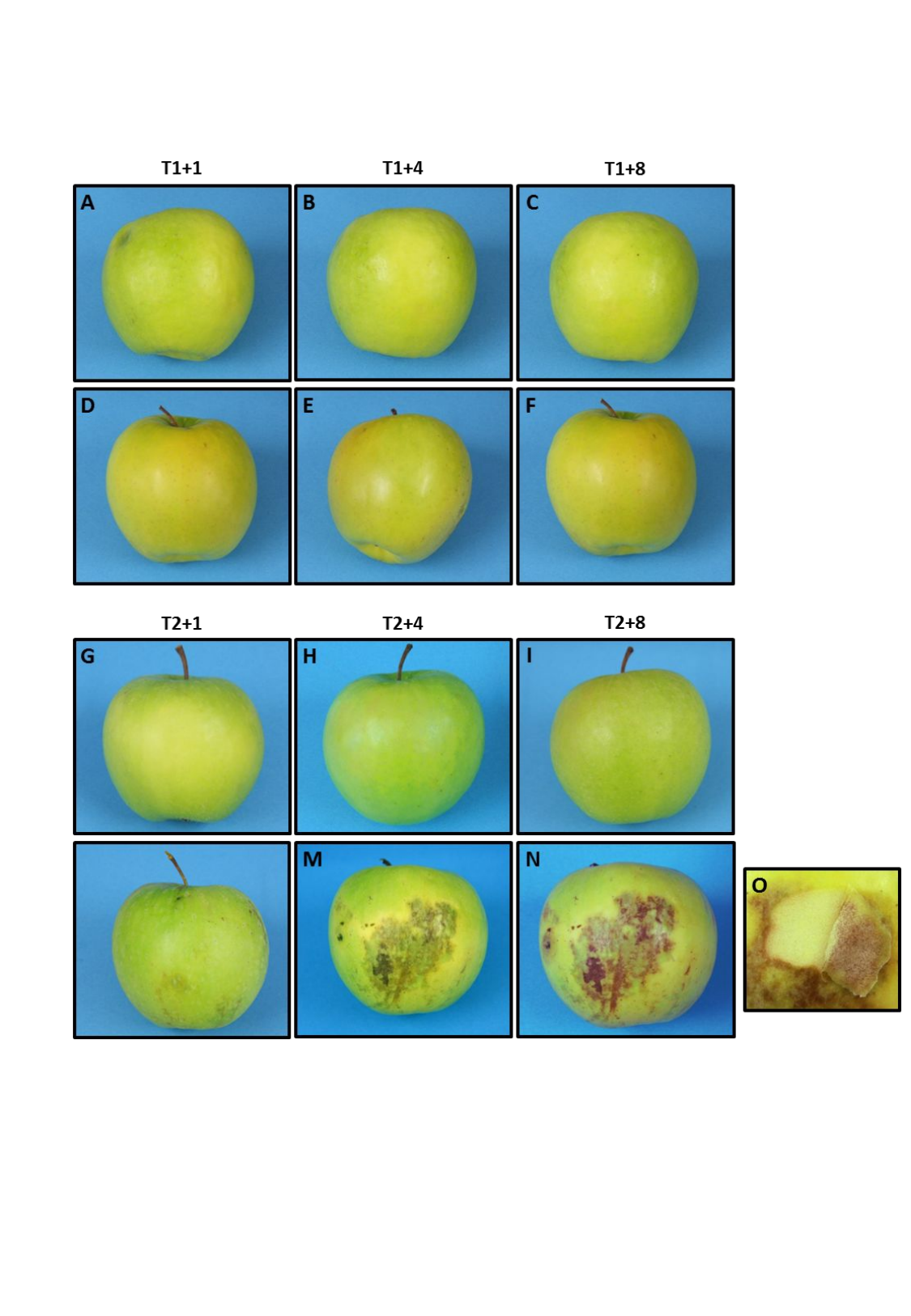

Supplement: Additional file 3: Figure S3. — Superficial scald development in “Granny Smith” apples. Representative pictures of superficial scald evolution in the control (CTR) and treated samples (1-MCP), after one (panel from A to F) and two months (panel from G to N) cold storage. Panel O shows a portion of scalded peeled apple tissue, at the T2 + 8 stage, highlighting isolation of the brown coloration in the skin alone. [file s12870-014-0193-7-S3.pptx]

## Slide 1
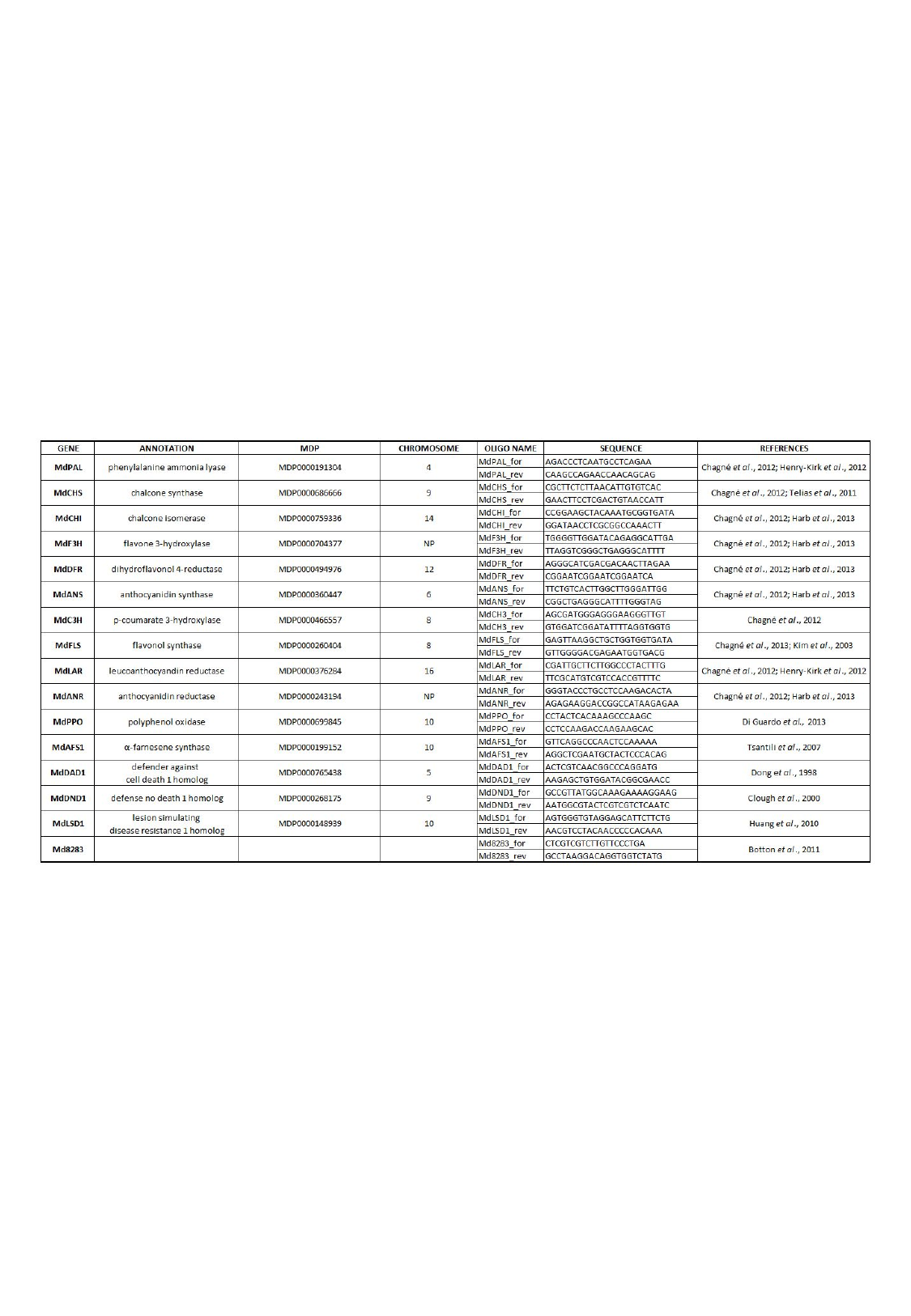

Supplement: Additional file 4: Table S1. — List of primers. List of all the primer pairs used in this work. The gene name, the annotation, the gene ID according to the code used in the apple genome database (www.rosaceae.org), the chromosome on which the gene is located, primer sequences designed for qRT-PCR analysis and the relative references are indicated for each pair. [file s12870-014-0193-7-S4.pptx]

## Slide 1
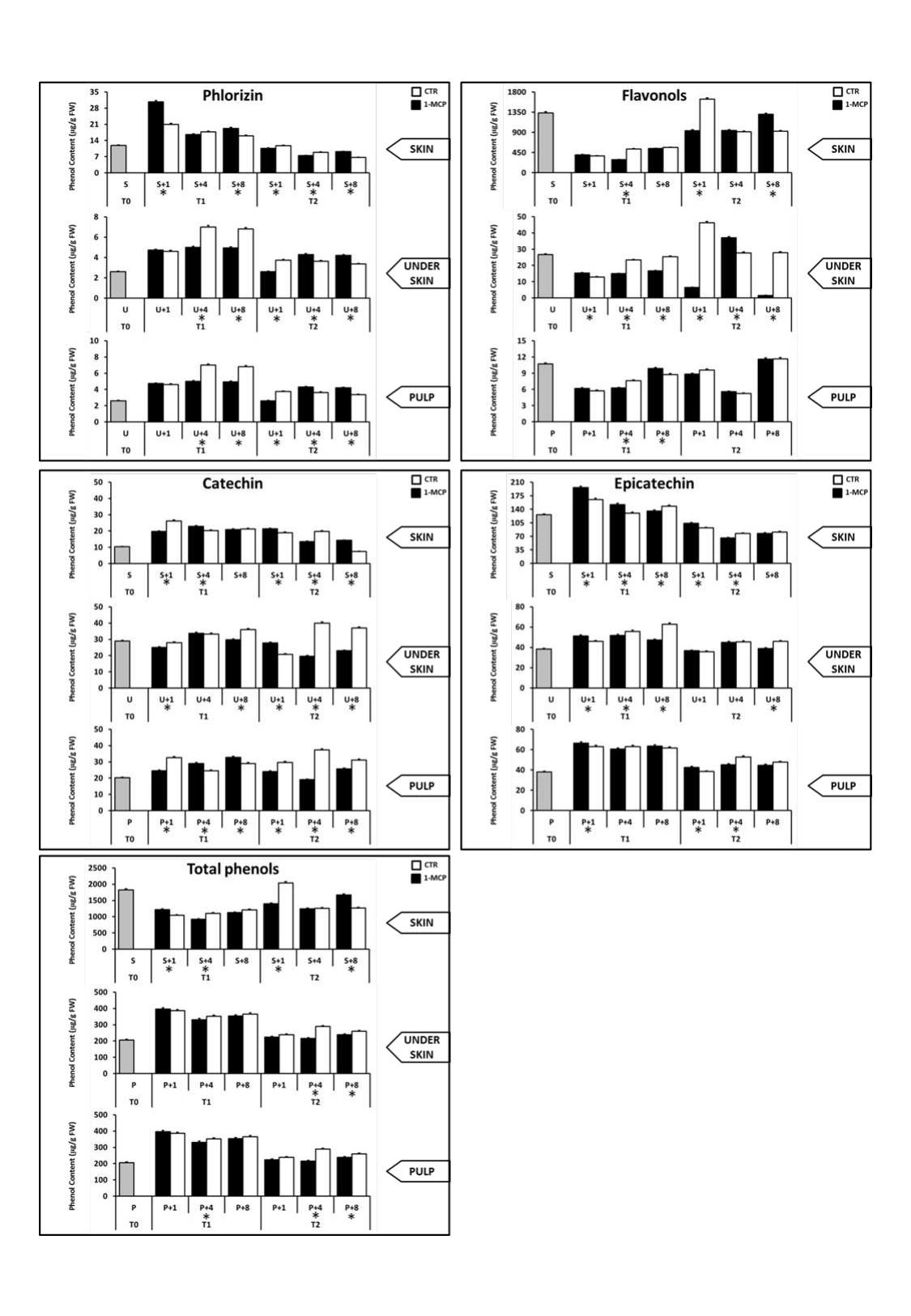

Supplement: Additional file 5: Figure S4. — Polyphenolic compounds characterization. Characterization of four major classes of polyphenolic compounds, namely phlorizin, flavonols, catechin and epicatechin. The last panel instead shows the general phenolic accumulation profile, including all the categories investigated in this study. The amount of each compound is expressed as μg/g of fresh weight (FW). The standard error is also reported for each bar. Asterisk indicates a difference statistically significant based on a LSD-ANOVA (P-value ≤ 0.05). [file s12870-014-0193-7-S5.pptx]

## Slide 1
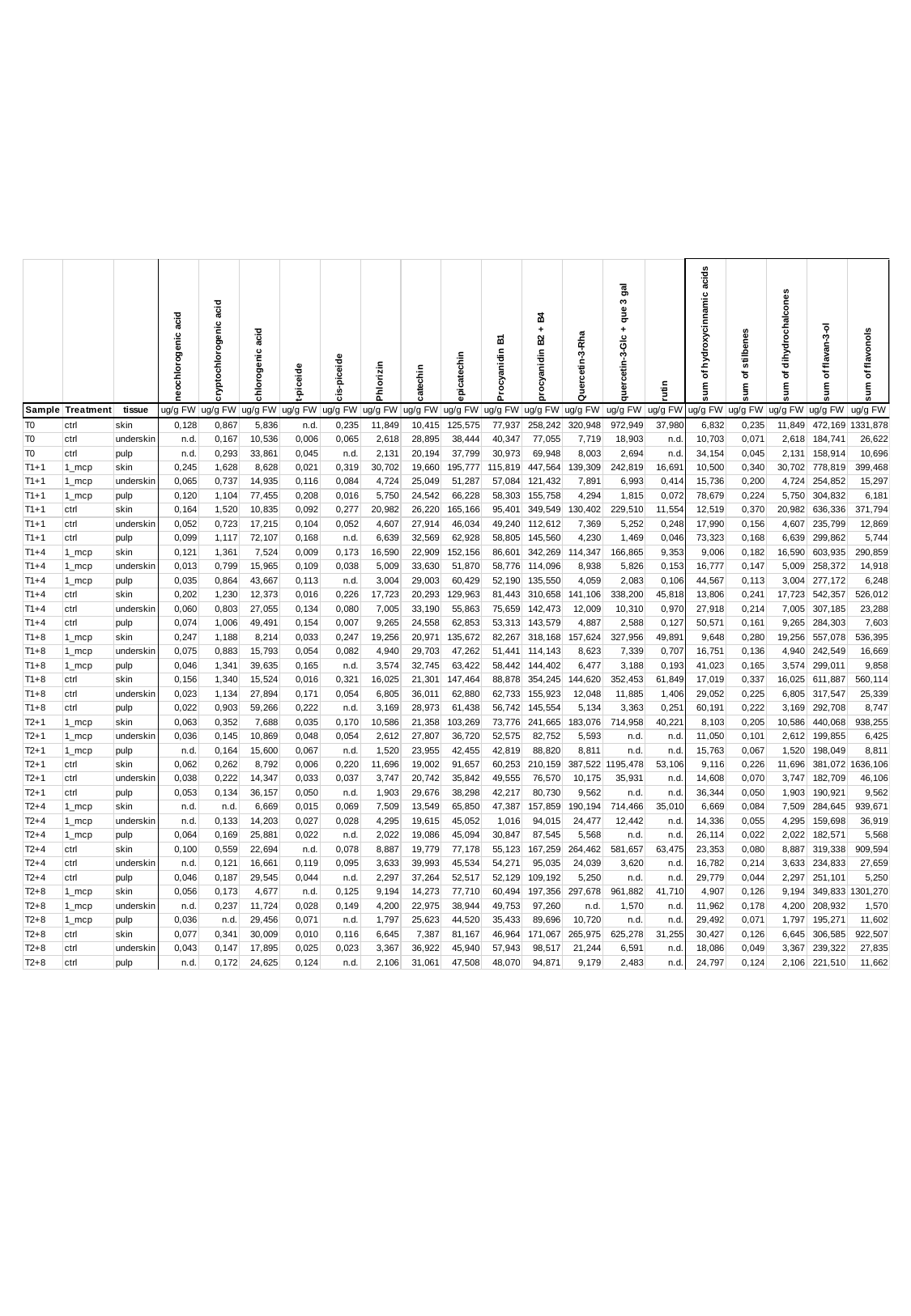

Supplement: Additional file 6: Table S2. — Table of polyphenol compounds quantified in this work. The amount of each compound is expressed as μg/g of fresh weight (FW). Each sample was represented by three separated biological replicates. [file s12870-014-0193-7-S6.pptx]

## Slide 1
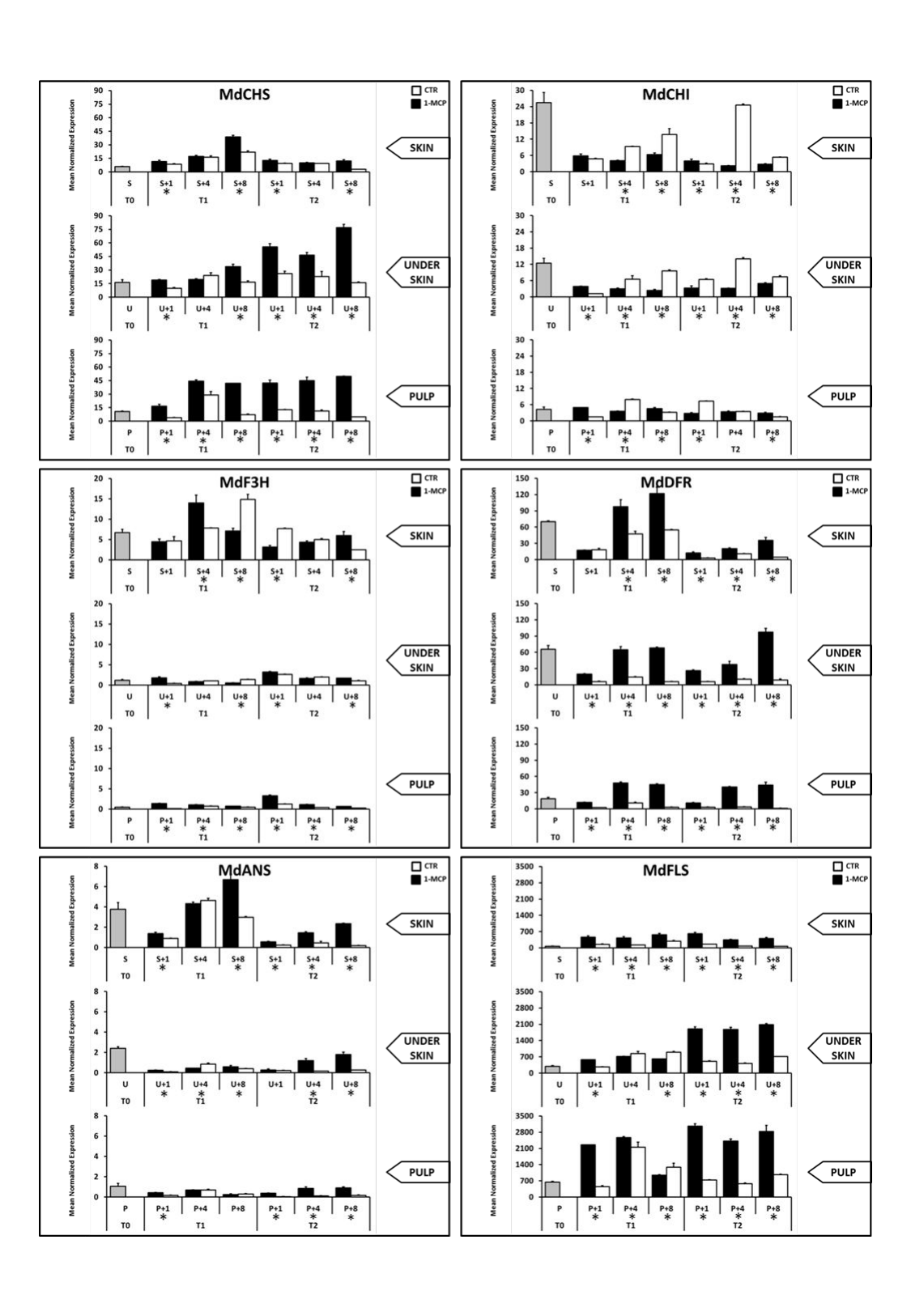

## Slide 2
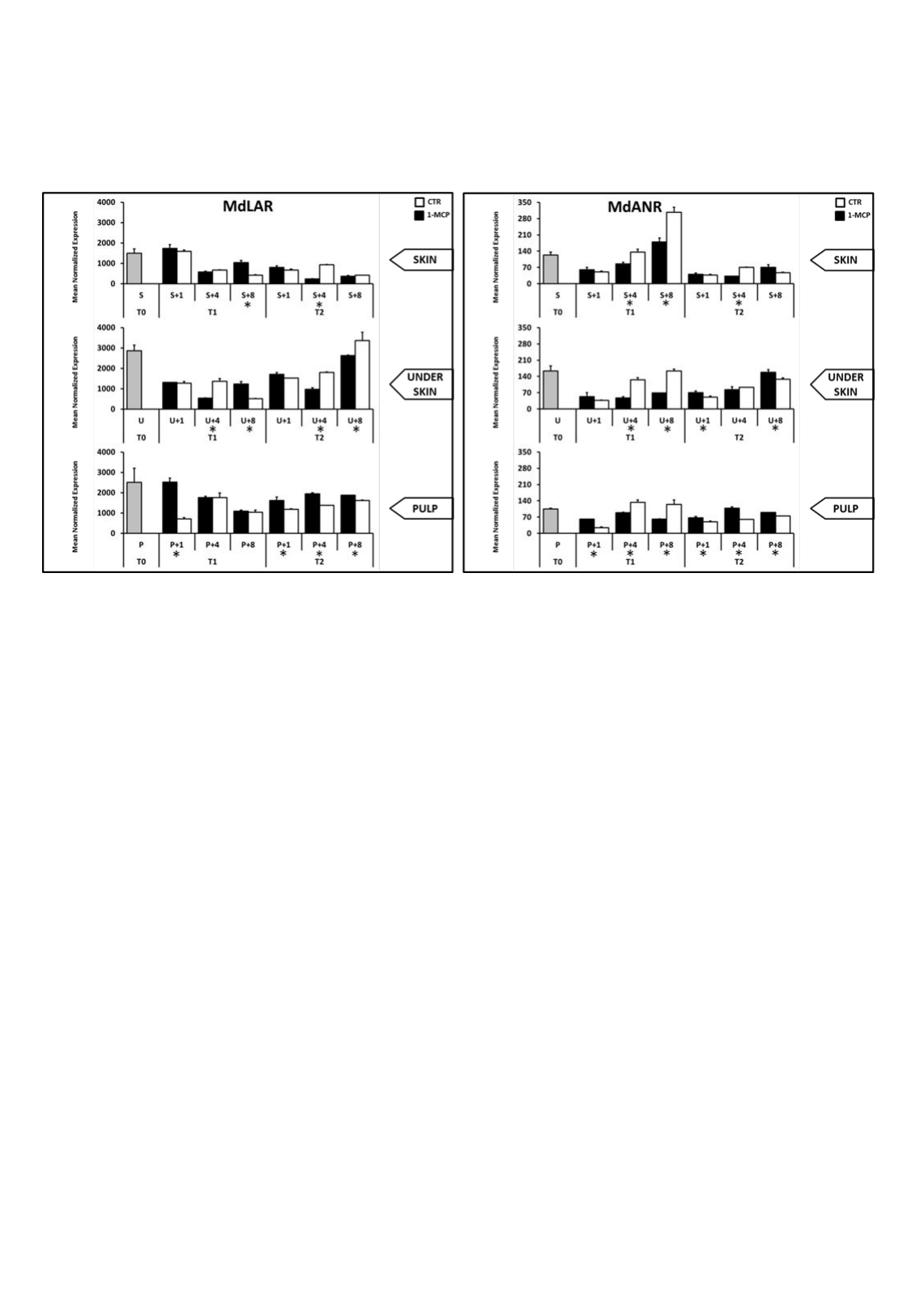

Supplement: Additional file 7: Figure S5. — Expression profile of genes involved in polyphenolic biosynthesis. Expression profile of all other genes participating in polyphenolic biosynthesis and not presented in the main text, namely MdCHS, MdCHI, MdF3H, MdDFR, MdANS, MdFLS, MdLAR and MdANR. The standard error is also reported for each bar. Asterisk indicates a difference statistically significant based on a LSD-ANOVA (P-value ≤ 0.05). [file s12870-014-0193-7-S7.pptx]
